# Using genomic databases to determine the frequency and population-based heterogeneity of autosomal recessive conditions

**DOI:** 10.1016/j.gimo.2024.101881

**Published:** 2024-08-03

**Authors:** William B. Hannah, Mitchell L. Drumm, Keith Nykamp, Tiziano Pramparo, Robert D. Steiner, Steven J. Schrodi

**Affiliations:** 1 Department of Genetics and Genome Sciences, Case Western Reserve University, Cleveland, OH; 2 Invitae Corporation, San Francisco, CA; 3 Travere Therapeutics, Inc, San Diego, CA; 4 Department of Pediatrics, University of Wisconsin School of Medicine and Public Health, Madison, WI; 5 Department of Medical Genetics, University of Wisconsin-Madison, Madison, WI

**Keywords:** Disease prevalence, Genetic epidemiology, Mendelian disorders, Recessive, Sequence database

## Introduction

The principles of the Hardy-Weinberg equilibrium (HWE) inform the frequency of genotypes within populations.^1^^,^^2^ Over time, meaningful real-world applications of HWE have emerged. In recent years, there has been an uptick in the number of publications evaluating the frequency of genetic conditions by applying principles of HWE to data primarily obtained from large publicly available genomic DNA sequence databases. Given the nuanced methods and the use of these studies to influence therapeutic development and public policy, a review of key considerations is of importance for physicians and other health professionals, geneticists, genetic counselors, statisticians, and public health experts among other specialists. The methods and analysis, interpretation of results, and implications differ across studies and conditions investigated. Here, we succinctly discuss the purpose and goals of such studies and highlight important differences in methods used in previous work, as well as limitations of various approaches. We also suggest important considerations in the attempt to harmonize approaches, methods, data analysis, interpretation, and terminology in future studies to promote best practices. Consistency across studies will facilitate understanding and promote accurate interpretation and conclusions.

### Overview of HWE and estimates of the frequency of genetic conditions

The use of large genomic databases to estimate the frequency of highly penetrant, monogenic conditions relies on application of the HWE.^1^^,^^2^ For a gene associated with an autosomal recessive condition, the individual frequencies of disease-causing (pathogenic) variants can be summed and then squared. Provided that the variants are rare and in linkage equilibrium (ie, variants are independent) this value represents the expected frequency of biallelic combinations of pathogenic variants at the time of conception when all assumptions are met. Note that in this commentary, the term biallelic describes a state of either homozygosity for a variant or variants present in compound heterozygosity. Similarly, HWE principles can be utilized to estimate the carrier frequency for a single site or the frequency of carrying a chromosome with 1 or more pathogenic variants. Equations for conception incidence and carrier frequency of autosomal recessive conditions are provided in the Supplemental Methods. Important assumptions, which may not always apply in real populations, are made when relying on HWE. These assumptions include random mating, infinite (or very large) population, no migration, no newly occurring variants, and no natural selection. Although no population exactly fits these assumptions, HWE has remarkable utility in genetic studies. HWE is applied in these cases because it provides an easily analyzed simplified model of a population, thereby providing a way to focus on epidemiologic matters important to genetic conditions. In essence, these methods approximate the frequency of a genetic condition by first estimating the expected number of individuals with the condition assuming the same age distribution and ancestry as the large genomic database used to inform the calculation. Notably, this procedure yields an estimate of the true frequency in the population. Improved estimates for some applications would include factoring in embryonic lethality, penetrance, and age of onset for the condition being analyzed. These factors, however, are often difficult to incorporate into the estimation procedure because they require parameters which are often unknown.

Additionally, it is important to note that there is a distinction between an individual identified with a laboratory diagnosis (for instance, biallelic pathogenic variants in a gene associated with an autosomal recessive condition, diagnostic biochemical testing, etc) and an individual diagnosed based on clinical manifestations. Incomplete penetrance, clinical misdiagnosis, a late age of onset, or variable expressivity with some unrecognized phenotypes may cause a discrepancy between estimates of the frequency of individuals with biallelic pathogenic variants and the frequency of individuals with a clinical diagnosis.

### Terminology

Further, terminology applied by authors of recent publications differs between studies that evaluate the frequency of genetic conditions based on data from genomic databases. For many real-world applications of these data, an understanding of the prevalence of a condition is most pertinent; when considering frequency of a condition for purposes of newborn screening birth prevalence may be the most relevant term. For very early onset, completely penetrant conditions, we would expect birth prevalence and incidence to be equal. For late onset conditions, birth prevalence is expected to be 0, whereas incidence will depend on the age distribution of the population. Pertinent terminology is highlighted in Table 1, including assumptions made when applying data derived from genomic databases to estimate these values. Oftentimes, the square of the sum of pathogenic allele frequencies in a gene associated with an autosomal recessive condition is used as an estimate of prevalence because prevalence is most germane to a number of real-world applications of these data. Although, it is important to be mindful of the assumptions made in this estimate. Assumptions may differ depending on whether the calculation is presented as an estimate of prevalence, birth prevalence, or incidence. Inasmuch as a condition deviates from the assumptions made, additional study limitations exist. In this commentary, the term frequency may be used when referring generically to how common a condition is observed. More granular terms (conception incidence, birth prevalence, incidence, and prevalence) will otherwise be used as appropriate.

**Table 1:** Pertinent terminology when considering the frequency of genetic conditions

Term	Definition	Considerations
Conception incidence	Proportion of individuals with biallelic variants at the time of conception. Most precisely, the square of the sum of minor allele frequencies in a gene associated with autosomal recessive inheritance is an estimate of conception incidence.	Conception incidence is essentially a theoretical construct. It is difficult, if not impossible, to diagnose a condition at conception.
Birth prevalence	Proportion of live births affected with a condition. Calculated by newborn screening studies that evaluate the proportion of newborns affected with a condition.	Differs from conception incidence in conditions with embryonic lethality or incomplete penetrance at birth.
Incidence	The number of new cases of a condition in a period of time.	For very early onset, completely penetrant inherited conditions, incidence equals birth prevalence.
Prevalence	Proportion of a population affected with a condition.	Differs from birth prevalence in conditions with shortened life expectancy or conditions lacking very early onset of symptoms.
Genetic Prevalence	Frequency of a fully pathogenic combination of alleles at the point of conception assuming no bias in the union of gametes.	The estimated proportion of a population that should have a genotype for a genetic disorder, irrespective of penetrance, age of onset, and other factors affecting the disease prevalence.

Studies that utilize genomic databases to estimate the frequency of autosomal recessive conditions often use the square of the sum of allele frequencies as an approximation of prevalence. This could be a limitation of the study when the condition is characterized by embryonic lethality, shortened life expectancy, or late onset of the condition.

The term chosen to express the frequency of a genetic condition will depend on the purpose of the estimate. For most real-world applications, prevalence will usually be the most relevant nomenclature; for newborn screening purposes, birth prevalence may be most pertinent. Although, it is important to be mindful of the assumptions made for the term used.

### Importance of estimating the frequency of conditions

Characterizing the frequency of rare genetic conditions may seem similar to an academic exercise without significant impact, but this is far from the case. Knowing the frequency of a genetic condition can have clinical utility with regard to weighing the likelihood of different conditions in a differential diagnosis. The likelihood of underdiagnosis of a particular condition can be identified.^3^^,^^4^ In the pharmaceutical industry, an understanding of the prevalence of a rare condition is essential and guides fundamental decision making in drug development, as well as commercial planning. Additionally, prevalence of a genetic condition is an important element that is considered during variant classification performed by genomic diagnostic laboratories.^5^ The birth prevalence of a rare condition is one important factor to weigh when considering the conditions to be included in newborn screening programs.^6^^,^^7^ In short, studies aimed at describing the frequency of rare conditions can inform scientists, pharmaceutical companies, and funding agencies involved in therapy development; elucidate underdiagnosis prompting changes in diagnostic approaches; aid in genetic variant classification; and influence public policy decisions, for example, in newborn screening. Finally, determining the frequency of conditions in low income countries and in populations underrepresented in biomedical research can influence policy to shift resources to groups adversely affected by rare genetic conditions. There are diverse real-world applications of determining the frequency of genetic conditions, and the accuracy of such estimates is critical for resource allocation and diagnostic approaches.

### Historic perspective

In discussing the application of HWE to genomic database data to estimate the frequency of genetic conditions, it is important to consider the context, namely, that other methods have been used to approximate the frequency of genetic conditions. Historically, numbers have sometimes been quoted for frequency of a condition based on very limited information. Even when references are provided to support the frequencies quoted, sometimes there is a lack of clarity as to how estimates were established because of incomplete method description, unclear results presentation, and/or study limitations. Significant knowledge gaps in the frequency of rare genetic conditions persist and likely accurate estimates are available for only a fraction of these conditions.^8^ In some cases, previous estimates have proven inaccurate based on new real-world observational data that inform more accurate determinations of frequency of a condition. As one example, with the advent of newborn screening, data in some areas suggest that Pompe disease may be more common than traditionally thought, based on retrospective analysis of carrier frequency data.^9^ Furthermore, as data emerge, our understanding may expand regarding how the frequency of genetic conditions differs between individuals of different ancestry.

Before the availability of large genomic databases, and still today, different methods have been utilized to estimate the frequency of genetic conditions. For example, the prevalence of glycogen storage disease type I (GSD I) is often cited as approximately 1 in 100,000. This was derived from the portion of GSD I among all GSD cases in 2 cohorts and the estimated prevalence of GSDs broadly.^10^ In some instances, the heterozygote frequency of a specific variant was determined and the incidence of a condition extrapolated. As one example, the frequency of the most common *DHCR7* variant (IVS8-1G>C) was determined in a population by sequence analysis of 1600 newborn screening dried blood spots. Based on the relative frequency of this variant among all disease-causing variants, the incidence of Smith-Lemli-Opitz Syndrome was inferred.^11^ Notably, in another example, it was suggested that cerebrotendinous xanthomatosis may be more common than was historically thought based on heterozygote frequency of a specific variant among healthy individuals of European ancestry. The variant was identified in 1 of only 115 individuals, highlighting the limitations of sample size in such studies.^12^ Since large genomic databases have become available, there have been numerous studies aimed at estimating the frequency of genetic conditions. As discussed later, other methods for estimating the frequency of genetic conditions without the use of genomic databases are also being applied.

### Introduction of large genomic databases and their utility in estimating the frequency of genetic conditions

The aggregation of genomic data from diverse studies into large, publicly available databases, such as the Exome Aggregation Consortium (ExAC) database,^13^ the genome aggregation database (gnomAD; the name given to the ExAC database after the addition of genome sequencing data),^14^ the UK Biobank,^15^^,^^16^ and the All of Us Research Program^17^ has had far-reaching benefits to the genetics community. These databases provide allele frequencies from large numbers of individuals by pooling data. Among the diverse uses of these databases, one application of these data has been to inform the use of variant frequency codes when classifying genetic variants.^18^ One review of the use of population databases for variant classification discusses the use of allele frequency, constraint, expression by base, and the co-occurrence of variants (informing haplotypes).^19^ Of course, allele frequencies often vary among individuals of different ancestries. Large genomic databases stratify allele frequency data by ancestry on the basis of principal component analysis on genome-wide polymorphisms with respect to reference populations; at times, self-reported ancestry may be incorporated. Principal component analysis clusters may be trained on self-provided ancestry labels. Such stratification of data may also allow for studies relevant to individuals of specific ancestries. It is considered good practice to not consider variants as pathogenic for monogenic disorders if the variant segregates at high frequency in one or more subpopulations. Indeed, a variant is benign if it is seen above a set threshold in at least 1 subpopulation. An important note is that in clinical practice, the diagnosing physician typically has access only to self-reported ancestry of the patient; therefore, by some study methods, estimating the likelihood of a particular condition with varying prevalence across ancestries will depend on self-reported information from patients. Finally, genomic databases are deficient in data from many underrepresented populations.

Large genomic databases have also proven useful in estimating the frequency of genetic disorders by making use of allele frequencies in the gene(s) of interest and principles of HWE. A number of such studies have been published recently, thereby expanding our epidemiologic understanding of Mendelian conditions. For perspective, some examples are highlighted in Table 2,^3^^,^^4^^,^^20^, ^21^, ^22^, ^23^, ^24^, ^25^, ^26^, ^27^, ^28^, ^29^ including some of the key findings. Availability of these databases has greatly facilitated epidemiologic research of rare genetic conditions, but challenges remain in the application of sound principles and calculations to infer the frequency of a condition based on the frequency of heterozygosity for a pathogenic allele. Additionally, there are important limitations, including small numbers of variants included in some analyses, determining which variants to include in the analysis based on evidence of pathogenicity, varying allele frequencies across populations, pregnancy losses and stillbirths, knowledge of genotype penetrance, and the possibility of analyzed variants being in linkage disequilibrium, among other limitations. Additionally, there may be an incomplete compilation of disease-causing variants. We note that when it is well understood what portion of a genetic condition has an unknown genetic etiology, the fraction of disease without a known genetic cause can be accounted for in the prevalence estimates.

**Table 2:** Examples are summarized of studies that used genomic databases to estimate the frequency of genetic conditions

Author and Citation	Genetic Condition	Database	Brief Summary of Findings
Appadurai et al^4^ (2015)	Cerebrotendinous xanthomatosis	ExAC	CTX is underdiagnosed and improvements in screening are needed.
Lanktree et al^20^ (2018)	Autosomal dominant polycystic kidney disease and autosomal dominant polycystic liver disease	gnomAD and BRAVO	A greater than anticipated prevalence of ADPKD causing variants were identified, and loss-of-function variants in genes associated with ADPLD were more common than anticipated. The possibility of undiagnosed individuals, incomplete penetrance, and a broadening phenotype were discussed.
Carter et al^21^ (2019)	Lysosomal acid lipase deficiency	gnomAD for allele frequencies. MEDLINE and EMBASE for prior prevalence estimates and variants.	LAL-D is very rare with lowest prevalence in individuals of East Asian, South Asian, and Finnish ancestry.
de Andrade et al^22^ (2019)	Li-Fraumeni syndrome	gnomAD. Additional databases to compare variant classification (ClinVar, HGMD, UMD_TP53).	The study estimated a prevalence of germline, pathogenic/likely pathogenic *TP53* variants that was greater than expected. It was noted that the estimates may not represent the prevalence of classical LFS.
Gao et al^23^ (2019)	Wilson disease	gnomAD. To identify prior prevalence estimates, MEDLINE and EMBASE were used.	Prevalence of Wilson disease may exceed prior estimates.
Vasiljevic et al^24^ (2019)	Zellweger spectrum disorder	ExAC	Incidence estimates approximate newborn screening estimates.
Borges et al^25^ (2020)	Mucopolysaccharidoses	ExAC and gnomAD	Overall prevalence estimated to be higher than published literature possibly because of underdiagnosis or limitations in variant classification. Prevalence calculated was similar to diagnosis-based studies of prevalence.
Weber Hoss et al^26^ (2020)	Classical Homocystinuria (HCU)	gnomAD	HCU incidence in Europeans was lower than reported in articles that evaluated small populations from northern Europe but similar to estimates based on newborn screening.
Gibson et al^27^ (2021)	X-linked, autosomal recessive, and digenic Alport syndrome and thin basement membrane nephropathy	gnomAD. Strategy confirmed using EVS and TOPMed.	Frequencies of *COL4A3*, *COL4A4*, and *COL4A5* were provided to suggest frequencies of Alport syndrome. Limitations acknowledged to include that penetrance must be accounted for, as well as variants not included in the analysis.
Park^28^ (2021)	Pompe disease	gnomAD	The predicted genetic prevalence exceeded earlier estimates, was consistent with newborn screening studies, and varied by ancestry.
Hannah et al^29^ (2022)	Primary ciliary dyskinesia	gnomAD. Data from collaborating diagnostic laboratory.	PCD may be more common than previously thought and may be more common among individuals of African ancestry. Genes most commonly implicated in PCD may vary depending on ancestry and may differ from genes traditionally described as most common.
Pramparo et al^3^ (2023)	Cerebrotendinous xanthomatosis	gnomAD. ClinVar and HGMD were utilized to identify pathogenic variants, and VarSome was utilized to inform variant classification.	This study supported the possibility of CTX underdiagnosis and highlighted the need for better detection. Prevalence was highest in the Asian population and lowest in the Finnish population.

For each study, the database(s) used and a brief description of findings are included. This table is intended to display only a sample of pertinent publications and is not a comprehensive list.

*ADPKD*, autosomal dominant polycystic kidney disease; *ADPLD*, autosomal dominant polycystic liver disease; *EVS*, Exome Variant Server; *HGMD*, Human Gene Mutation Database.

There are important differences in the methods authors have applied in studies utilizing large genomic databases to estimate the frequency of genetic conditions. There have been varying strategies concerning selection of variants to include in the calculations, methods used to calculate the frequency of a genetic condition, inclusion of different ancestral groups in the analysis, and the calculation of confidence intervals. When HWE principles are applied, complete penetrance is usually assumed in calculating frequency, an assumption that does not hold true for some genetic conditions. Note that if incomplete penetrance is adequately quantified for a condition, this could be accounted for when using genomic databases to estimate frequency of a condition. When conception incidence is used as a proxy for prevalence, assumptions are also made that there is no embryonic lethality, that there is very early onset of the condition, and that life expectancy is not shortened. Additionally, there are inherent statistical limitations to the point estimate of conception incidence. Typically, the square of the sum of pathogenic allele frequencies is used. This is an approximation to the more accurate form that incorporates the (very) small probability that 2 or more pathogenic alleles are carried on 1 chromosome. The form incorporating this co-occurrence of pathogenic alleles on a homolog takes the form of the square of 1 minus the product of 1 minus the pathogenic allele frequencies. In addition, there are differences in the general statistical estimation approach. Under a frequentist estimation procedure, the allele frequencies are treated as fixed values and the point estimate results from applying HWE and the corresponding 95% confidence interval typically relies on a Gaussian approximation.^21^^,^^23^^,^^25^^,^^28^ This is an easily implemented approach to estimating the conception incidence but can have small errors in the procedure. Alternatively, a Bayesian approach to estimating the posterior probability density and credible intervals of prevalence estimates has been applied.^30^ The Bayesian approach treats allele frequencies as random variables informed by the counts of the alleles and the sample size. The Bayesian estimation procedure therefore is reliant on the posterior probability of biallelic variants (conditioned on the allelic counts and sample size). The point estimate is then the expectation of this posterior probability and the credible set is calculated through integration of the tails of the posterior density.

### Components to consider when estimating the frequency of a genetic condition using genomic databases

Given different approaches in past studies, we suggest minimum essential components as guidelines for investigators to consider as future studies are planned that use genomic databases to estimate the frequency of genetic conditions. These components are listed briefly in Table 3, and more detailed discussion of nuanced considerations are outlined in the Supplemental Methods. The suggested components attempt to harmonize approaches, methods, data analysis, interpretation, and terminology in future studies.

**Table 3:** Suggested guidelines for some essential elements that should be considered in future studies that use genomic databases to estimate the frequency of genetic conditions

Component	Brief Description
Description of variant inclusion and exclusion criteria	The criteria for determining which variants are included in the study analysis should be detailed clearly in methods. This is among the most critical considerations for study design.
Selection of appropriate populations	Allele frequencies and disease rates vary substantially across ancestries and populations. The selection of populations that reflect the conclusions drawn from the analyses is essential.
Choice of database	Differences in variant content may be found across databases and across versions of the same database. In addition to differences in the populations included in the databases, there may be differences in the way sequencing data are analyzed and in how variants are called. Filtering steps may also vary between databases. Numerous factors can be considered when investigators choose the database that is best fit for a specific study.
Discussion of penetrance, age of onset, and survival	Inasmuch as it is known for the condition studied, a discussion of penetrance and the spectrum of age of onset would be useful. If penetrance is well characterized, this could be accounted for in calculations to estimate the frequency of the condition. Age of onset and early mortality are important to note when this information is well understood and contextualized based on the characteristics of individuals included in the genomic database.
Consideration of terms used to describe the frequency of a condition	For future studies, terms used to describe the frequency of a condition should be considered thoughtfully (ie, incidence, birth prevalence, or prevalence) including the assumptions made when using these terms (Table 1).
Estimating frequency of genetic conditions with locus heterogeneity	If the frequency of a condition with multiple causative genes is estimated, then the frequencies calculated for each individual gene may be summed. If it is well described what portion of the genetic condition can be diagnosed by analysis of the genes included in the study (sensitivity of genetic testing), this can be factored into calculations.
Statistical analysis	Whether estimating frequency of a genetic condition by a frequentist estimation or a Bayesian approach, statistical analysis should be applied to determine confidence intervals or credible intervals.
Acknowledging study limitations	Possible study limitations should be considered thoughtfully and comprehensively discussed. Possible limitations may include methods for determining variants to include in analysis, deviations of the genetic condition from HWE assumptions, deviations from assumptions made in terminology for frequency of a condition, and absence of disease-causing variants from genomic databases, among other limitations.

Additionally, in individuals affected clinically, sometimes only a single heterozygous variant can be identified on genomic testing because there are limitations in the sensitivity of testing. Likewise, as discussed above, disease-causing variants may not be present in large genomic databases and may not be accounted for in these studies. It is also important to recognize that estimates may be biased and higher in populations for which clinical variation has been most intensively studied. When basing calculations on specific ancestries, be mindful of how many individuals of that ancestry are represented in the genomic database.

### Other methods for estimating the frequency of genetic conditions

This commentary is focused on the application of large genomic databases to analyze the epidemiology of genetic conditions. There are other methods for evaluating epidemiology of genetic conditions that are beyond the scope of this article. These approaches include evaluation of newborn screening data,^9^^,^^31^, ^32^, ^33^ mining of electronic health records for pertinent International Classification of Diseases codes,^34^ manual review of health records,^35^ evaluation of survey data,^36^ analysis of insurance claims,^37^, ^38^, ^39^ and review of registry data,^40^^,^^41^ among other methods. Different results for estimating the frequency of genetic conditions have been acknowledged based on different methods. As an example, prevalence estimates of Wilson disease differ depending on whether it is based on review of clinical records or registries or frequency of *ATP7B* variants^42^ (penetrance has been suggested as an explanation for the difference between Wilson disease prevalence estimates based on genetic versus clinical data^43^). Given the breadth of methods aimed at estimating the frequency of genetic conditions and the increasing use of genomic databases in epidemiologic studies, this manuscript focused on approaches utilizing such databases, but many of the definitions, statistical approaches, and recommendations for best practices outlined are applicable to other methods.

A tool is being developed by investigators at Broad Institute to streamline the estimation of genetic prevalence of conditions leveraging allele frequency data from gnomAD (https://genie.broadinstitute.org). This tool is designed to calculate the estimated genetic prevalence of rare recessive conditions through the squared sum of the pathogenic variants and does so across several populations. Such a tool can provide investigators with a platform to aid in determining and sharing work on developing genetic prevalence estimates.

### Closing thoughts

We have concentrated on estimates of prevalence among autosomal recessive conditions. As individuals heterozygous for variants in genes associated with autosomal recessive conditions typically do not exhibit severe clinical manifestations, the representation of these individuals in large population-based genomic databases is thought to be relatively unbiased. This likely yields more accurate estimates of frequency for autosomal recessive conditions compared with other models of inheritance, such as dominantly acting traits for which there may be a reduced representation of dominant pathogenic variants in population-based genomic databases. We acknowledge a limited body of literature that has evaluated conditions with other inheritance patterns. This includes the use of gnomAD to estimate the prevalence of germline *TP53* variants,^22^ as well as a study that ranked carrier frequencies of autosomal recessive and X-linked disorders.^44^ Another study evaluated carrier frequencies in specific ancestral groups of genes associated with autosomal recessive and X-linked conditions in using gnomAD with implications for curating screening panels.^45^ One study evaluated the frequency of variants in genes associated with Alport syndrome, which displays different inheritance patterns.^27^

It is a complex task to apply statistical approaches using large genomic databases to estimate the frequency of genetic conditions or the frequency of being heterozygous for a pathogenic allele. Our goal has been to discuss concisely some important considerations in such studies in the context of a commentary article. This is not a comprehensive discussion. There is an increasing number of such studies that the reader may find of interest. In addition to the references already included, genetic epidemiology studies using genomic databases include evaluation of polycystic kidney and liver disease,^20^ Wilson disease,^23^ lysosomal acid lipase deficiency,^21^ cystic fibrosis,^46^ and autosomal recessive mitochondrial disorders,^47^ among other publications. These studies are relevant to the discussion given the methods and study aims. It is thoroughly acknowledged that the references in this commentary should not be considered an exhaustive list of this rapidly growing body of literature. We encourage readers and investigators to be mindful of nuanced methods, the potential purpose and benefits of such studies, and the limitations inherent with certain protocols.

## Data Availability

This commentary does not include experimental data.

## Conflict of Interest

William B. Hannah was a consultant for PTC Therapeutics and ReCode Therapeutics. Mitchell L. Drumm has no disclosures relevant to the manuscript. Keith Nykamp is an employee and stockholder of Invitae Corporation. Tiziano Pramparo was a full-time employee of Travere Therapeutics, Inc at the time of manuscript development and submission and may have an equity or other financial interest in Travere Therapeutics, Inc. Robert D. Steiner is a consultant for Leadiant, Mirum, and PTC Therapeutics. Robert D. Steiner is an employee with equity of PreventionGenetics, part of Exact Sciences. Steven J. Schrodi has no disclosures relevant to the manuscript.
