# Supplementary material for: Using genomic databases to determine the frequency and population-based heterogeneity of autosomal recessive conditions
Source: Genet Med Open. 2024 Aug 3;2:101881. doi: 10.1016/j.gimo.2024.101881 (PMC11613865; doi:10.1016/j.gimo.2024.101881)
Supplement: Supplementary Data [file mmc1.pdf]

Equations for conception incidence and carrier frequency

Pertinent equations for the calculation of conception incidence and carrier frequency of autosomal recessive diseases are shown below.

$$Conception\ Incidence = \left[1 - \prod_{i=1}^{k}(1 - p_i)\right]^2 \approx \left(\sum_{i=1}^{k} p_i\right)^2$$

where there are k independent fully penetrant pathogenic variants at the gene and p_i is the allele frequency of the ith variant in a particular population exhibiting HWE.

The carrier frequency can be estimated by the following formula:

$$Carrier\ frequency = 2\left[\prod_{i=1}^{k}(1 - p_i)\right]\left[1 - \prod_{i=1}^{k}(1 - p_i)\right] \approx 2\left(\sum_{i=1}^{k} p_i\right)\left[1 - \left(\sum_{i=1}^{k} p_i\right)\right]$$

Components to consider when estimating disease frequency from genomic databases: expanded discussion

There are important considerations that warrant special attention when estimating disease frequency using genomic databases. Some are included briefly in Table 3, but it is not a comprehensive list. We have strived to capture what we consider to be the most important considerations. Below some important points are elaborated.

1. Determination of variants to include in the analysis. Among the most critical and challenging factors in the prevalence calculation is determining which variants to include in the analysis. Ideally, to obtain the most accurate estimates all disease-causing variants would be included with all variants that are not disease-causing excluded. However, often only variants that have been found in confirmed cases with well-characterized clinical features and/or biochemical markers can be deemed pathogenic with little uncertainty. Additionally, in a perfectly ideal situation all variants would be weighted by their penetrance when performing calculations, although for many diseases the penetrance of specific combinations of variants is not well-described and this is not currently feasible. In ClinVar, a star system is in place to convey the level of review for a particular variant. Expert panel or practice guideline designation can be achieved under criteria that include approval by the Clinical Genome Resource (ClinGen). The FDA has evaluated ClinGen expert curated variant data, and FDA recognized variants are in ClinVar. For many variants that are found in the large genomic databases, judgment must be applied based on the type of variant. The probability of being disease-causing is evaluated based on available scientific evidence and current guidelines. There are limitations in our ability to classify variants, and these limitations are inherent in prevalence estimates based on genomic databases. Some databases, such as ClinVar, can be cross-checked for variant classifications that have been entered by laboratories based on widely accepted guidelines from the American

College of Medical Genetics and Genomics (ACMG) and the Association for Molecular Pathology (AMP).[1] The exclusion of variants of uncertain significance (VUS) may lead to an underestimate of prevalence while inclusion would result in an overestimate. VUS can be excluded for a lower-end estimate and then included for an upper-end estimate, giving a range for prevalence estimate. Particular caution must be exercised on VUS with a relatively high frequency given their outsized impact on prevalence estimates.

Many variants that may be ascertained through genomic databases have not undergone rigorous classification based on guidelines. It can be difficult to determine which variants to include in the analysis when sifting through large numbers of variants in a genomic database that have not previously been classified. Of course, with limited clinical and functional data, there are significant limitations in how ACMG/AMP criteria can be applied to analyze variants. When variants have not been classified based on guidelines, there are tools that can be used to inform the likelihood of a variant being disease-causing. For instance, researchers can query variants in databases such as gnomAD on the basis of presumed loss-of-function (pLoF). Classically, the PVS1 code (very strong strength of evidence of pathogenicity) can be applied to pLoF variants (frameshift, nonsense, canonical splice, etc.) in genes known to be associated with disease via a loss of function mechanism. Likewise, the frequency in a genomic database of a variant in a gene of interest may inform the likelihood of the variant being disease causing, including application of codes PM2, BA1, or BS1. Some have proposed methods to establish a maximum credible allele frequency for a variant in a particular gene to be pathogenic. The information that contributes to such a determination may include the prevalence of disease associated with the gene, penetrance, and allelic heterogeneity.[2] As another example of data in genomic databases that can inform the likelihood of pathogenicity, in silico programs/predictors have been used for application of the PP3 or BP4 codes. Recent recommendations have provided guidance for when, and at what strength of evidence, to apply these codes in evaluating a variant.[3]

The codes discussed above were not established for the selection of variants to use in an analysis of disease prevalence; however, they may inform the likelihood of particular variants being disease-causing. The data that support these codes are present in genomic databases or other resources and could be of utility when determining which variants to include in an analysis of prevalence. One example of variants that may be included by some researchers would be a presumed loss-of-function variant (in a gene associated with disease due to loss-of-function) that is "rare" in a large database. Such a variant may be considered likely pathogenic with application of the pLoF code (PVS1) and the PM2 variant frequency code. Researchers may determine thresholds for excluding "common" variants. Computational evidence may provide additional evidence to inform which variants to include or exclude, and recent data support upgrading the strength of evidence in certain situations.[3] There are inherent limitations to applying evidence of pLoF, allele frequencies, and in silico programs when determining which variants to include and exclude in an analysis of disease prevalence. For instance, there are caveats to the use of PVS1 and considerations for downgrading.[4] Additionally, it may be difficult to establish gene-specific maximum credible allele frequencies for exclusion of "common" variants. Methods to determine a maximum credible allele frequency depend on an

understanding of disease prevalence, among other considerations. Indeed, the purpose of these studies is to estimate the prevalence of disease, so interpretive caution is recommended when using a threshold for variant inclusion that depends on knowledge of disease prevalence. If collaborating with a diagnostic laboratory that performs genetic testing, the laboratory's formal variant classifications can inform how to incorporate variants into the analysis.[5]

In summary, determining which variants to include and exclude from the analysis is one of the most difficult and fundamental considerations when using genomic databases to estimate disease prevalence. It is among the most critical factors affecting study accuracy, and it is important to be mindful of the limitations of variant classification in such studies.

2. Variants that may not be found in genomic databases. Some disease-causing variants may not be included in genomic databases, and this can be acknowledged in limitations of the study. For instance, intragenic copy number variants can constitute a significant portion of disease-causing variants in some conditions[6] but may be absent from gnomAD. In addition, large structural variants are poorly interrogated with short-read sequencing, and as such are often absent in variant repositories. Also, private variants may be so rare that they are not present in the database. Obviously, not all disease causing variants have been identified. Many ultra-rare variants may be absent related to the limited size of the database (number of individuals). Further, variants that contribute to individuals not being present in collection of genomic database samples (e.g., poor health) can represent a sampling bias.

   Recently, it has been proposed that one can obtain the total number of pathogenic variants by estimating the number of unobserved pathogenic variants through applying a conditional Poisson density which is conditioned on the observed pathogenic variants within a population-based sequenced sample set.[7]

3. Possibility of incomplete penetrance. When estimating conception incidence of autosomal recessive disease by squaring the sum of allele frequencies, complete penetrance is assumed. HWE more technically estimates the portion of conceptions with biallelic variants. When penetrance is complete and there is no embryonic lethality, this calculation also reflects birth prevalence. If the portion of individuals with biallelic disease-causing variants who are symptomatic is known, then the incomplete penetrance can be accounted for in the calculation to estimate the disease frequency. Incomplete penetrance will reduce the expected frequency of disease compared to the calculation under the assumption of fully penetrant alleles. Of note, genetic modifiers at other loci may affect penetrance, and this cannot be accounted for in such studies. When prevalence estimates based on genomic databases differ from real-world observational data, this could reflect, at least in part, incomplete penetrance of the disease or other limitations of study design.

4. Possibility of linkage disequilibrium of variants determined to be disease-causing. If multiple pathogenic variants are in linkage disequilibrium but included independently in the frequency calculation, this could result in an overestimate of the disease prevalence and incidence. Haplotypes containing more than one variant determined to be disease-causing may either be

accounted for in the methods, or the possibility of linkage disequilibrium may be acknowledged as a limitation. That said, population genetic models support the idea that rare pathogenic variants are likely to be close to linkage equilibrium given that they are typically very recently occurring variants on different haplotypes.

5. Possibility of genomic databases containing data from affected individuals (late-onset diseases, conditions with reduced penetrance) or related individuals. Data from related individuals or affected individuals could be in the genomic database, and this could be a study limitation. As these databases expand, they will likely become better representations of large populations.

6. Autosomal recessive conditions with locus heterogeneity. At times, the prevalence of rare diseases may be investigated when there are multiple genes associated with the condition. If the portion of disease related to each particular gene is known, or if the portion of disease related to all genes collectively included in the analysis is known, then this information can be taken into account when estimating the prevalence of disease.

7. Deviations from HWE. The use of HWE equations to estimate disease prevalence assumes, naturally, that the disease follows the assumptions of HWE. Many rare genetic conditions are caused by variants which disrupt functionally important specific regions of the genome including exons and regulatory motifs. These regions are more likely to be subjected to selective forces, modifying the standing variation in those regions. Other HWE assumptions such as the absence of *de novo* variants and genetic drift are violated. Of particular importance is the extent of consanguinity in the population studied. Small levels of inbreeding or assortative mating can dramatically increase the incidence of disease compared to random mating (although part of data quality control of genomic databases may include removing related individuals). Segregation distortion and uniparental disomy can also produce a bias in the correspondence between allele frequencies and HWE genotype frequencies. It is important to be mindful of possible deviations from HWE as a study limitation.

References

1. Richards S, Aziz N, Bale S, et al. Standards and guidelines for the interpretation of sequence variants: a joint consensus recommendation of the American College of Medical Genetics and Genomics and the Association for Molecular Pathology. *Genet Med*. May 2015;17(5):405-24. doi:10.1038/gim.2015.30

2. Whiffin N, Minikel E, Walsh R, et al. Using high-resolution variant frequencies to empower clinical genome interpretation. *Genet Med*. Oct 2017;19(10):1151-1158. doi:10.1038/gim.2017.26

3. Pejaver V, Byrne AB, Feng BJ, et al. Calibration of computational tools for missense variant pathogenicity classification and ClinGen recommendations for PP3/BP4 criteria. *Am J Hum Genet*. Dec 1 2022;109(12):2163-2177. doi:10.1016/j.ajhg.2022.10.013

4. Abou Tayoun AN, Pesaran T, DiStefano MT, et al. Recommendations for interpreting the loss of function PVS1 ACMG/AMP variant criterion. *Hum Mutat*. Nov 2018;39(11):1517-1524. doi:10.1002/humu.23626

5. Hannah WB, Seifert BA, Truty R, et al. The global prevalence and ethnic heterogeneity of primary ciliary dyskinesia gene variants: a genetic database analysis. *Lancet Respir Med*. May 2022;10(5):459-468. doi:10.1016/S2213-2600(21)00453-7
6. Truty R, Paul J, Kennemer M, et al. Prevalence and properties of intragenic copy-number variation in Mendelian disease genes. *Genet Med*. Jan 2019;21(1):114-123. doi:10.1038/s41436-018-0033-5
7. Bainbridge MN. Determining the incidence of rare diseases. *Hum Genet*. May 2020;139(5):569-574. doi:10.1007/s00439-020-02135-5
